# Serum Neutralization of Omicron BA.5, BA.2 and BA.1 in Triple Vaccinated Kidney Transplant Recipients

**DOI:** 10.1016/j.ekir.2022.12.004

**Published:** 2022-12-10

**Authors:** Rune M. Pedersen, Line L. Bang, Ditte S. Tornby, Anna C. Nilsson, Christian Nielsen, Lone W. Madsen, Isik S. Johansen, Thomas V. Sydenham, Thøger G. Jensen, Ulrik S. Justesen, the COVAC-TX study group, Jesper R. Davidsen, Mikael K. Poulsen, Rozeta Abazi, Lars Vitved, Yaseelan Palarasah, Claus Bistrup, Thomas E. Andersen

**Affiliations:** 1 Department of Clinical Microbiology, Odense University Hospital and Research Unit for Clinical Microbiology, University of Southern Denmark, Odense, Denmark; 2 Department of Clinical Immunology, Odense University Hospital and Research Unit for Clinical Immunology, University of Southern Denmark, Odense, Denmark; 3 Department of Infectious Diseases, Odense University Hospital and Research Unit for Infectious Diseases, University of Southern Denmark, Odense, Denmark; 4 Department of Cancer and Inflammation, University of Southern Denmark, Odense, Denmark; 5 Department of Nephrology, Odense University Hospital and the Nephrology Research Unit, University of Southern Denmark, Odense, Denmark

## Introduction

Kidney transplant recipients (KTRs) develop a lower-than-normal antibody (Ab) response against the COVID-19 mRNA vaccines and are consequently more vulnerable to breakthrough infections.^1^^,^^2^ Although booster vaccinations given to these patients increase serum-levels of spike Abs,^3^ SARS-CoV-2 variants have emerged with increasing vaccine-evading properties, the most recent being the Omicron variant of concern. The original Omicron lineage, BA.1 (also known as B.1.1.529), is neutralized less efficiently by booster vaccinated KTRs than the ancestral and the Delta variant, as demonstrated by authentic virus and pseudovirus neutralization assays conducted with blood from these patients.^4^, ^5^, ^6^ During spring 2022, Omicron BA.1 was rapidly replaced by the Omicron BA.2 subvariant in Europe and the United States and a descendant from Omicron BA.2, the even more transmissible Omicron BA.5 subvariant, which has now replaced other subvariants worldwide and to date (October 2022) continues to dominate the COVID-19 pandemic with more than 85% prevalence in most countries.^7^ BA.5 deviates from BA.2 by 4 additional mutations, including 2 in the receptor binding domain of the spike protein, which makes this subvariant even more immune-evasive in otherwise healthy individuals.^8^ Although the neutralization status in KTRs postvaccination against the original Omicron BA.1 was earlier estimated,^4^, ^5^, ^6^ the neutralization capacity in KTRs and solid organ transplanted recipients in general, against the currently dominating descendants of Omicron BA.1, remains to be elucidated.

Here, we report the spike Ab levels and serum neutralization capacity against isolates of Omicron BA.1, BA.2, and BA.5 in KTRs who have received 3 doses of the BNT162b2 Pfizer-BioNTech mRNA vaccines (*n* = 44); and compare these values to the BA.5 neutralization capacity in healthy controls at the same level of vaccination (*n* = 20). In addition, we report the SARS-CoV-2 reactive T-cell response in a subset of the KTRs (*n* = 25).

## Results

The KTRs in this cross-sectional study is part of a KTR cohort that has been described previously, together with the plaque reduction neutralization test (PRNT).^1^ The PRNT_90_, which indicates the serum titer that reduces the plaque forming ability of the virus by >90%, was measured. A titer of 10 was applied as the cut-off, indicating the lowest titer that yields actual neutralization in our assay, because a PRNT_90_ titer of 8.8 was recently shown to indicate the threshold of real-world protection against the Omicron variant of concern in humans.S1 Three clinical Omicron strains, BA.1, BA.2, and BA.5, and 1 Delta strain, were used for the PRNT_90_ (Genome sequences: GenBank accession no. ON055874 for BA.1, ON055857 for BA.2, OP225643 for BA.5, and ON055856 for the Delta strain). All experimental work with SARS-CoV-2 was conducted in approved biosafety level 3 facilities. The serum samples were also analyzed for spike Ab levels using the LIAISON SARS-CoV-2 TrimericS IgG assay (DiaSorin, Saluggia, Italy). Finally, we performed flow cytometry on freshly collected blood to quantify SARS-CoV-2 inducible T cells in 25 KTRs and 3 healthy controls (for methods, statistics, and patient flow chart, see Supplementary Cohort Data, Supplementary Methods, Supplementary Statistics, and Supplementary Figures S1 and S2).

**Figure 1:**
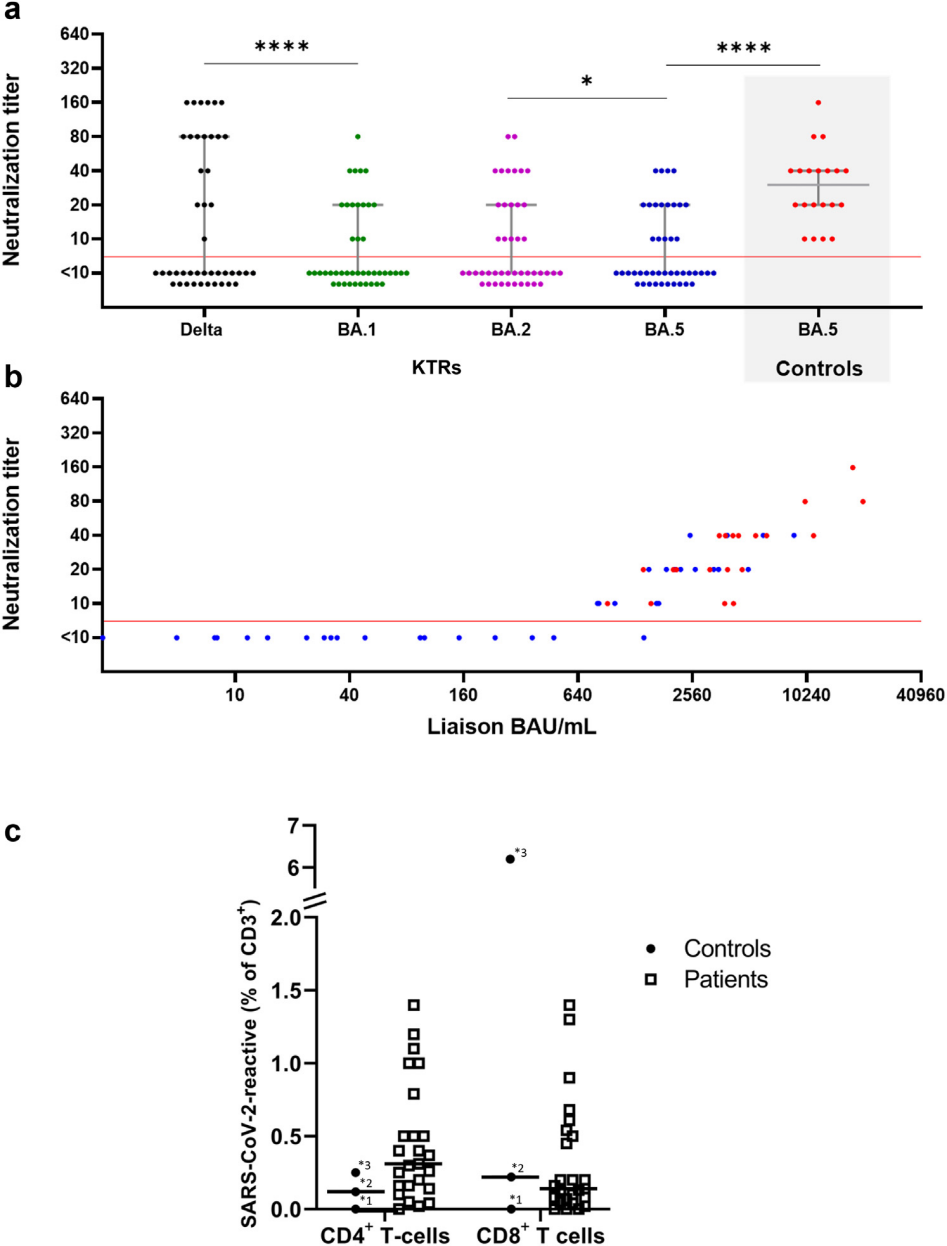
Neutralization of authentic SARS-CoV-2 Delta, Omicron BA.1, Omicron BA.2, and Omicron BA.5 strains, antibody levels and SARS-CoV-2 specific T cells among KTRs who have received 3 doses of the BNT162b2 vaccines. (a) Neutralization titers (PRNT_90_) of KTRs (*n* = 44) against the Delta, Omicron BA.1, Omicron BA.2, and Omicron BA.5; and neutralization titers against Omicron BA.5 of healthy controls (*n* = 20) of sera obtained 5 to 6 weeks after the third BNT162b2 vaccine dose. Overall, there were significant differences between the PRNT_90_ values for 2 or more of the tested SARS-CoV-2 subvariants (*P* < 0.0001, Friedman test). Significant differences as calculated in the subsequent one-to-one subvariant comparison are indicated with asterisks. Medians and IQRs are indicated for the individual groups by gray bars. Horizontal red line indicates the neutralization threshold. (b) Dot plot showing spike Ab levels as measured with the LIAISON SARS-CoV-2 TrimericS IgG assay in relation to the neutralization titers of the BA.5 strain by sera from KTRs (blue dots) and healthy controls (red dots). Overall, the summed-up Ab levels of the KTRs and the healthy controls correlated with the neutralization titer (*r* = 0.91, Spearman’s rank correlation, *P* < 0.0001). Ab levels are shown in BAU/ml. The manufacturer provided Ab threshold is 34.8 BAU/ml. Horizontal red line indicates the neutralization threshold. (c) The proportion of total CD4+ and CD8+ T cells inducible by SARS-COV-2 S-protein peptides measured in a subpopulation of the KTRs (*n* = 25, open squares) and 3 healthy controls (black dots). The healthy controls are ∗1: a naïve person with respect to both vaccination and infection; ∗2: a person who had received 2 mRNA vaccinations, and ∗3: an infection convalescent person who after recovery had received 2 mRNA vaccinations (for more details, see Supplementary Material). Median values are indicated by horizontal lines. A negative control subject is included who is both COVID-19 and COVID-19 vaccination naïve (black dots). ∗*P* = 0.05-0.01, ∗∗∗∗*P* < 0.0001. Ab, Antibody; BAU, binding antibody units; COVID-19, coronavirus disease 2019; IQR, interquartile range; KTR, kidney transplant recipient; PRNT90, 90% plaque reduction neutralization test; SARS-CoV-2, severe acute respiratory syndrome coronavirus 2.

The PRNT_90_ was performed on serum collected at a median of 38 days (interquartile range [IQR] 33−49) after the third BNT162b2 vaccination, from 44 KTRs (19 females, 25 males) with a median age of 60 years (IQR 51–67). As a reference for BA.5 neutralization, PRNT_90_ was also performed on serum collected at a median of 42 days (IQR 41–42) after the third BNT162b2 vaccination, from 20 healthy control subjects (12 females, 8 males) with a median age of 57 years (IQR 50−60). The characteristics of the KTR cohort are shown in Table 1. We found that 19 of 44 (43%), 15 of 44 (34%), 18 of 44 (41%) and 17 of 44 (39%) of the KTRs displayed above threshold neutralization of the Delta, Omicron BA.1, Omicron BA.2, and Omicron BA.5 strains, respectively (Figure 1a). The PRNT_90_ titer range of the KTRs toward the different strains were as follows: Delta <10 to 160, Omicron BA.1 <10 to 80, Omicron BA.2 <10 to 80, and Omicron BA.5 <10 to 40; with titer levels toward Omicron BA.5 being significantly lower for the KTRs than for the healthy controls (*P* < 0.0001, Mann-Whitney *U* test) (Figure 1a). The median Ab levels in sera from the KTRs as measured on the Liaison platform was 193 Binding Antibody Units (BAU)/ml (IQR 8−1743), whereas the median Ab level of the controls was 4115 BAU/ml (IQR 2943−5800). This difference was statistically significant (*P* < 0.0001, *t*-tests). Ab levels correlated with the PRNT_90_ titer of Delta (*r* = 0.88, *P* < 0.0001, Spearman’s correlation [SC]), Omicron BA.1 (*r* = 0.82, *P* < 0.0001, SC), Omicron BA.2 (*r* = 0.86, *P* < 0.0001, SC), and Omicron BA.5 (*r* = 0.86, *P* < 0.0001, SC) among the KTRs as well as with Omicron BA.5 among the controls (*r* = 0.74, *P* = 0.0002, SC) (Figure 1b; Supplementary Figure S3). Finally, cellular immunity was evaluated in a subset of KTRs (*n* = 25) and in 3 healthy controls using T cell flow cytometry. This analysis showed that 21 of 25 KTRs (84%) had detectable levels of cytotoxic CD8+ T cells directed against the SARS-CoV-2 spike protein, and 24 of 25 KTRs (96%) had spike protein-reactive CD4+ T helper cells. The KTRs had similar levels of T helper cells compared to both of the vaccinated controls, and similar levels of cytotoxic T cells compared to the 2-times vaccinated, infection-naïve control. The infection convalescent, 2-times vaccinated control had considerably higher levels of cytotoxic T cells than all others, indicating the significant boost in these cells upon infection (Figure 1c).

**Table 1:** Characteristics of the kidney transplant recipient cohort according to neutralization response against BA.5 after the third dose of the BNT162b2 (Pfizer-BioNTech) vaccine

Demographic characteristics	BA.5 neutralizers	BA.5 non-neutralizers	*P*-value
Numbers (%)	17 (39)	27 (61)	N/A
Age Y (IQR)	54 (47–63)	63 (58-–73)	0.04
Female (%)	8 (47)	11 (41)	0.76
BMI (IQR)	27.5 (23.7–31.6)	27.4 (23.9–30.0)	0.51
TX characteristics			
Time from TX Y (IQR)	7.6 (4.9–12.0)	6.0 (2.4–15.0)	0.95
TX number			0.83
First TX (%)	14 (82)	19 (70)	-
Second TX (%)	3 (18)	7 (26)	-
Third TX (%)	0 (0)	1 (4)	-
Deceased donor (%)	9 (53)	16 (59)	0.76
Induction			
Rituximab (%)	1 (6)	0 (0)	N/A
Anti-CD25 (%)	11 (65)	15 (55)	0.75
Anti-CD25 + Rituxmab (%)	1 (6)	1 (4)	N/A
Thymoglobuline (%)	3 (18)	6 (22)	N/A
Thymoglobuline + Rituxmab (%)	1 (6)	5 (19)	N/A
Maintenance			
Tacrolimus (%)	16 (94)	19 (70)	0.12
Tacrolimus CO ng/ml (IQR)	5.3 (4.9–6.2)	5.3 (4.9–5.9)	0.62
Ciclosporin A (%)	1 (6)	5 (19)	N/A
Ciclosporin A CO nmol/l (IQR)	355	471 (277–551)	N/A
Everolimus (%)	0 (0)	0 (0)	N/A
MMF/MPA (%)	14 (82)	26 (96)	0.28
MMF (%)	9 (53)	21 (77)	0.11
MPA (%)	5 (29)	5 (19)	0.47
aMMF/MPA: fraction of full dose (IQR)	1.0 (0.67–1.00)	0.75 (0.67–1.0)	0.44
MMF/kg (IQR)	17.0 (11.3–21.0)	17.1 (14.0–21.3)	0.50
MPA/kg (IQR)	12.4 (6.2–16.2)	7.7 (6.2–11.2)	0.32
Azathioprine (%)	3 (17)	1 (3)	N/A
Azathioprine mg (individual dosings)	25–50–100	75	N/A
Steroids (%)	1 (6)	4 (15)	N/A
Plasma creatinine μmol/l (IQR)	106 (97–167)	158 (105–207)	0.09
eGFR ml/min (IQR)	59 (33–71)	39 (23–57)	0.06
Underlying disease			0.91
bNonimmune disease (%)	7 (41)	10 (37)	-
cImmune disease (%)	7 (41)	8 (30)	-
Diabetes mellitus (%)	1 (6)	3 (11)	-
Infection (%)	1 (6)	2 (7)	-
Unknown (%)	1 (6)	4 (15)	-

BMI, body mass index; CO, concentration in plasma; eGFR, estimated glomerular filtration rate; IQR, interquartile range; MMF, mycophenolate mofetil; MPA, mycophenolic acid; N/A, not applicable; TX, transplant.

Continuous variables are presented in medians and IQR. Binomial variables are presented in numbers and percentages. Differences were analyzed with the *t*-test and Fisher exact test. We have not performed comparisons for data with n < 10, which have been designated N/A.

a Full dose of MMF is 1000 mg bid except in patients treated with tacrolimus then full dose is 750 mg bid. Full dose MPA is 720 mg bid except in patients treated with tacrolimus then full dose is 540 mg bid.

b Non-immune disease designates diseases such as cystic kidney diseases, Alports disease, urinary outlet obstruction etc.

c Immune disease designates diseases such as glomerulonefritis, systemic lupus, ANCA associated vasculitis etc.

## Discussion

Our results show that despite the splitting of the original Omicron variant of concern into increasingly transmissible subvariants during 2022, KTRs boosted with the BNT162b2 vaccine remain at approximately the same neutralization levels regardless of the Omicron subvariant tested. Compared to the distribution of SARS-CoV-2 PRNT_90_ titers among healthy persons after 3 dosesS2 and PRNT_90_ titers in the current KTR cohort after the second BNT162b2 dose,^1^ the KTRs’ PRNT_90_ titers after the third dose now appears to divide into 2 subpopulations, namely a group of non-neutralizers that remains below PRNT_90_ threshold, and a neutralizing group which has responded well to the booster by raising neutralization capacities to levels close to the healthy control group. Looking at the Ab levels, many of the non-neutralizers generate detectable levels after the third (booster) vaccination with measurable spike Abs in 36 of 44 (82%) of the KTRs compared to only 28 of 57 (49%) after the second vaccination.^1^ With respect to the KTR’s T cell response, we found that 84% of the KTRs had measurable levels of both cytotoxic and T helper cells, which were inducible by the SARS-CoV-2 spike protein. T cell responses against coronaviruses are typically more long-lived than Ab responses against this virus, and coronavirus specific T cells are critical in the protection against severe disease.^9^

In conclusion, our results indicate that the KTRs’ neutralization capacity against the currently dominating Omicron BA.5 approximates their ability to neutralize earlier Omicron subvariants. Moreover, a considerable proportion of the KTRs show a relatively robust neutralization response against all tested Omicron subvariants after the first BNT162b2 booster. In addition, we observed a tendency toward a division of KTRs into low responders and high responders, with the first group, despite a general increase in Ab levels, still being unable to neutralize recent SARS-CoV-2 variants. Additional boosters including the now available bivalent mRNA vaccines may increase Ab levels and affinities even in this group, thus providing an important fundamental level of protection against currently circulating SARS-CoV-2 strains.

## Disclosure

All the authors declared no competing interests.

## Appendix

### List of members of the COVAC-TX study group

Jesper R. Davidsen, Mikael K. Poulsen, Rozeta Abazi

## Supplementary Materials

**Supplementary Methods.**

**Supplementary Statistics.**

**Supplementary Cohort** **Data** **.**

**Supplementary References.**

**Figure S1.** Flow cytometry gating strategy.

**Figure S2.** Patient flow chart showing the inclusion of kidney transplant recipients for plaque reduction neutralization test and T cell analysis. NC, nucleocapsid.

**Figure S3.** Neutralization of authentic SARS-CoV-2 Delta, Omicron BA.1 and Omicron BA.2 strains in relation to spike antibody levels.
