# Supplementary material for: Serum Neutralization of Omicron BA.5, BA.2 and BA.1 in Triple Vaccinated Kidney Transplant Recipients
Source: Kidney Int Rep. 2022 Dec 10;8(3):667–71. doi: 10.1016/j.ekir.2022.12.004 (PMC9741493; doi:10.1016/j.ekir.2022.12.004)
Supplement: Supplementary File (PDF) [file mmc1.pdf]

Serum neutralization of Omicron BA.5, BA.2 and BA.1 in triple vaccinated kidney transplant recipients

Rune M. Pedersen, Line L. Bang, Ditte S. Tornby, Anna C. Nilsson, Christian Nielsen, Lone W. Madsen, Isik S. Johansen, Thomas V. Sydenham, Thøger G. Jensen, Ulrik S. Justesen, the COVAC-TX study group, Lars Vitved, Yaseelan Palarasah, Claus Bistrup, Thomas E. Andersen.

Supplementary material

Cohorts

The Kidney transplant recipients (KTRs) in this cross-sectional study were part of a larger cohort of solid organ transplant recipients followed with measurements of SARS-CoV-2 specific antibody levels at several time points following COVID-19 vaccination [S3]. The KTRs in this study (n=44) were invited for participation since they had previously participated in another cross-sectional study regarding neutralization capacity following their second BNT162b2 vaccination [S4]. Here, the same KTRs and healthy controls (see below) were invited for serum collection four to six weeks after their third BNT162b2 vaccination (for patient flowchart, see Figure S2). A group of 20 age-matched healthy volunteers (12 females, 8 males) was included as a control group for the assessment of KTRs' BA.5 neutralization. Clinical and laboratory data were extracted from the patients' medical records. The Regional Committees on Health Research Ethics for Southern Denmark (Record no. 77786, ID S-20210007C) approved the study. All patients signed informed consent and could withdraw their consent at any time.

Supplementary methods

Immunoassays

Serum samples were analysed for spike antibodies (Abs) using the LIAISON® SARS-CoV-2 TrimericS IgG assay (Diasorin S.p.A, Italy) according to manufacturer's recommendations. Serum samples from the KTRs were tested for antibodies against the nucleocapsid (NC) with a qualitative CMIA SARS-CoV-2 IgG assay (Abbott Laboratories). Results >1.4 AU were interpreted as NC positive (manufacturer defined) and excluded due to suspicion of previous SARS-CoV-2 infection.

SARS-CoV-2 isolates

Three clinical SARS-CoV-2 Omicron strains (BA.1, BA.2 and BA.5) and one SARS-CoV-2 Delta strain were used for Plaque Reduction Neutralization Test (PRNT). These strains were genome sequenced using the ARTIC network nCoV-2019 sequencing protocol v3 (LoCost) and V3 primers, on a MinION sequencing instrument (Oxford Nanopore Technologies, Oxford, UK) [S5]. Sequences are available at GenBank, accession no. ON055874 for BA.1, ON055857 for BA.2, OP225643 for BA.5 and ON055856 for the Delta strain.

Plaque reduction neutralization assay

The PRNT used in this study is regarded as the "gold-standard" of measuring the neutralizing Ab activity and correlates with clinical protection from COVID-19 [S6, S7]. In brief, SARS-CoV-2 viral stock, kept at -80°C, was incubated with serially diluted patient serum and subsequently used to infect precultured Vero E6 cells (for details, see [S4]). Plaque forming units (pfu) were counted and the serum dilution yielding more than 90% reduction in pfu identified (90% plaque reduction neutralization test, hereafter PRNT90 [S8]). A predefined cut-off of 10 was applied, which has been shown to reflect the actual neutralization threshold in humans [S1]. All experimental work with SARS-CoV-2 was conducted in approved biosafety level 3 (BSL-3) facilities (license no. 20200016905/5).

T cell flow cytometry

Twenty-five of the KTRs and three healthy controls were included for analysis of SARS-CoV-2 specific T cell response. Briefly, KTRs within a limited geographical distance from the collection site were invited for participation four to six weeks after the third BNT162b2 dose. The healthy controls were 1) a naïve person (female) with respect to both vaccination and infection; 2) a person (female) who had received two BNT162b2 doses, and 3) a person (male) who was convalescent from infection with the ancestral SARS-CoV-2 strain and had subsequently received two mRNA 1273 (Moderna) vaccinations.

Blood specimens were collected from in heparin tubes (BD Vacutainer, Becton Dickinson, UK) and stored for 16-20 hours at 4°C. Mononuclear cells were purified by density gradient centrifugation using the SepMate^{TM} -50 PBMC isolation kit (Stemcell Technologies, catalog #85450) according to the manufacturer's instructions. 10^6 cells were transferred to wells in a 96-well flat-bottom microtiter plate and stimulated by 1) SARS-CoV-2 S-protein peptides (PepTivator SARS-CoV-2 Prot_S Complete); 2) CytoStim for positive control; 3) water/10% DMSO for negative control. Following 2 hours incubation, brefeldin A was added and incubated for 4 hours. Cells were then harvested and stained for 10 min using Viobility 405/452 Fixable Dye, then fixed and permeabilized. Finally, detection of SARS-CoV-2-reactive CD4+ and CD8+ T lymphocytes was performed by adding antibodies included in the SARS-CoV-2 T Cell Analysis Kit for PBMC's (Miltenyi Biotec, 130-128-156). The cells were analyzed on a FACSAria III (Becton-Dickinson) flow cytometer. Doublets, dead cells, debris, CD14+ and CD20+ cells were excluded. Following pregating on CD3, and CD4 and CD8, respectively, cytokine and activation markers were assessed for the cells: CD154 and TNF-α for CD4+ cells; TNF-α and IFN-γ and for CD8+ cells (see supplementary figure S1 for gating strategy).

Supplementary figures

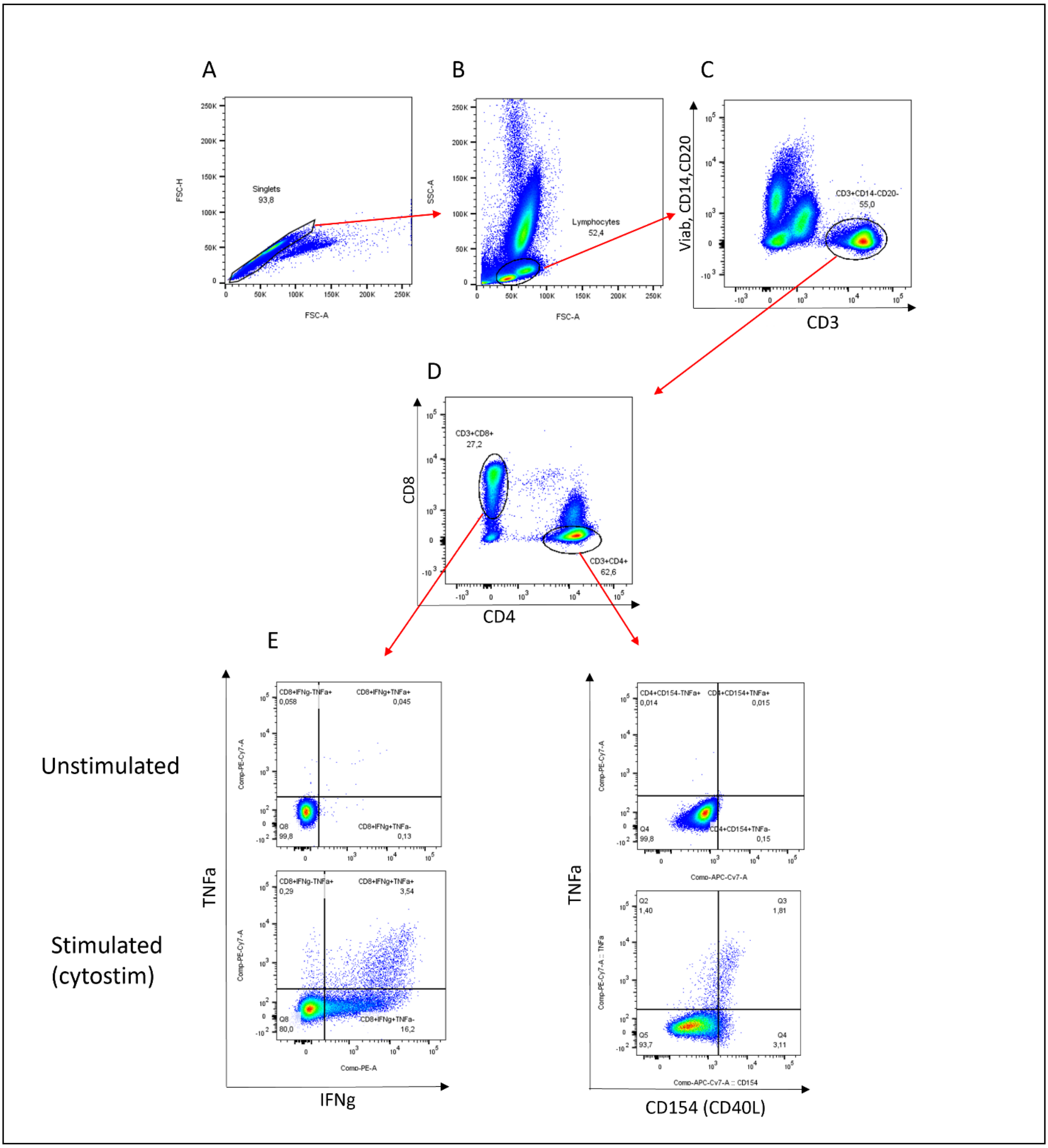

Supplementary Figure S1: flow cytometry gating strategy.

A-B. Cells were first selected as singlets on the basis of FSC-A and FSC-H parameters. B-C. Lymphocytes displaying low FSC-A and low SSC-A physical parameters were identified. C-D. T cells

by CD3 positivity and dead cells, CD14+, CD20+ were excluded by a dump channel. D-E. Identification of activated CD4+ and CD8 + was done using CD154 and TNF-α for CD4+ cells; TNF-α and IFN-γ and for CD8+ cells.

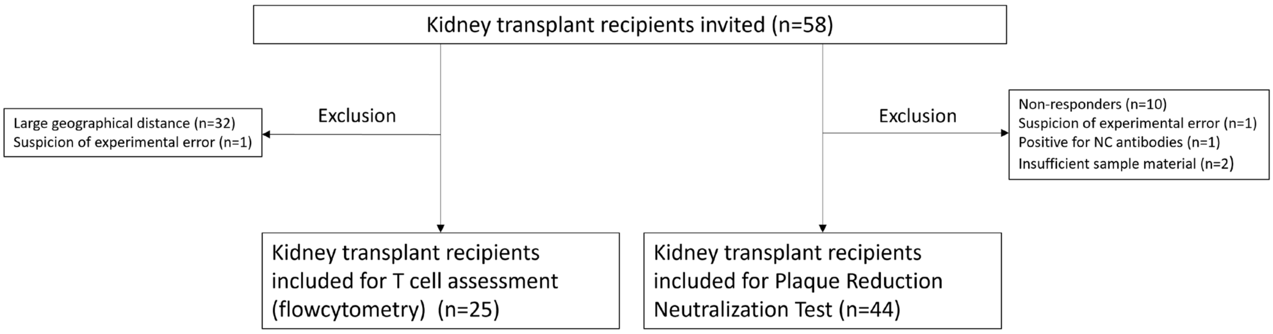

Supplementary Figure S2. Patient flow chart showing the inclusion of kidney transplant recipients for Plaque Reduction Neutralization Test and T cell assessment. Abbreviations: NC, nucleocapsid.

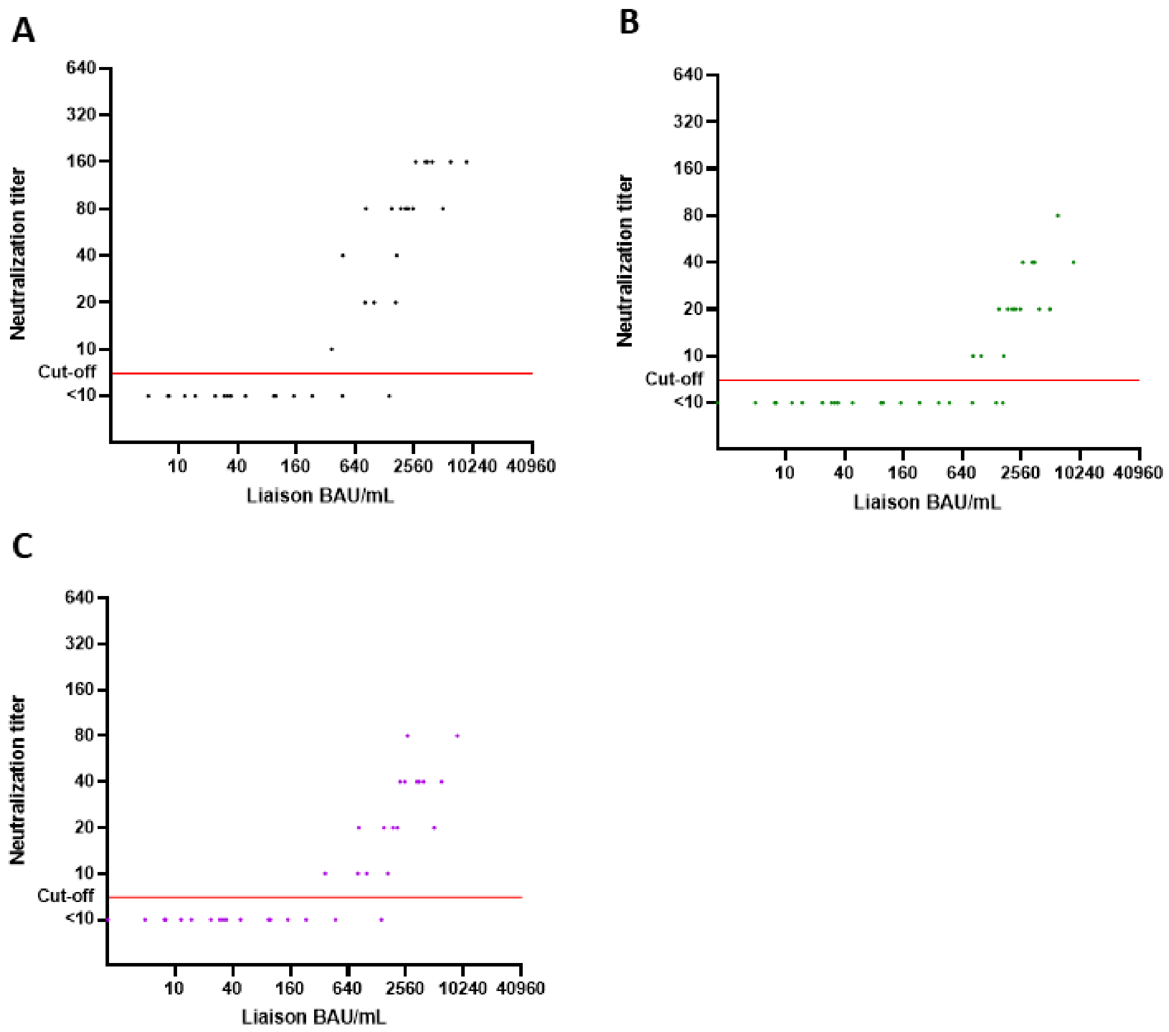

Supplementary figure S3. Neutralization of authentic SARS-CoV-2 Delta, Omicron BA.1 and Omicron BA.2 strains in relation to spike antibody levels. Dot plots of spike Ab levels as measured with the LIAISON® SARS-CoV-2 TrimericS IgG assay shown in relation to the PRNT90 titers of Delta (A), Omicron BA.1 (B), and Omicron BA.2 (C) using sera from 44 KTRs. Ab levels are shown as BAU/mL. Manufacturer-provided Ab threshold is at 34.8 BAU/mL. Red lines indicate neutralization threshold levels. Abbreviations: Ab, antibodies; BAU, binding antibody units; IgG, immunoglobulin G; KTR, kidney transplant recipient; PRNT90, 90% plaque reduction neutralization test; SARS-CoV-2, severe acute respiratory syndrome coronavirus 2.

Statistics

Graphical presentations and estimations of the Spearman's rank correlation coefficient (ρ) were performed with GraphPad Prism software version 9.1.2. (San Diego, CA). Neutralization titers were initially compared with the Friedman test to account for the multiple comparisons problem. Subsequently, Wilcoxon signed-rank test were used for one-to-one comparisons and Mann Whitney test for comparison between KTRs and controls using GraphPad Prism. The Friedman test, Student's t-test and Fisher's exact test were conducted using the STATA software version 17.0 (College Station, TX). All data are presented with medians and $p<0.05$ is considered statistically significant.

Supplementary references

S1. Cheng SMS, Mok CKP, Leung YWY, et al. Neutralizing antibodies against the SARS-CoV-2 Omicron variant BA.1 following homologous and heterologous CoronaVac or BNT162b2 vaccination. Nat Med 2022;28:486-489.

S2. Pedersen RM, Bang LL, Tornby DS, et al. Omicron BA.5 Neutralization among Vaccine-Boosted Persons with Prior Omicron BA.1/BA.2 Infections. Emerging Infectious Diseases 2022;28(12):10.3201/eid2812.221304.

S3. Balsby D, Nilsson AC, Möller S, et al. Determinants of Antibody Response to a Third SARS-CoV-2 mRNA Vaccine Dose in Solid Organ Transplant Recipients: Results from the Prospective Cohort Study COVAC-Tx. Vaccines (Basel). 2022;10(4):565.

S4. Pedersen RM, Bang LL, Tornby DS, et al. The SARS-CoV-2-neutralizing capacity of kidney transplant recipients 4 weeks after receiving a second dose of the BNT162b2 vaccine. Kidney Int 2021;100:1129–1131.

S5. Tyson JR, James P, Stoddart D, et al. Improvements to the ARTIC multiplex PCR method for SARS-CoV-2 genome sequencing using nanopore. bioRxiv 2020, doi: 10.1101/2020.09.04.283077.

S6. Lau EHY, Tsang OTY, Hui DSC, et al. Neutralizing antibody titres in SARS-CoV-2 infections. Nat Commun 2021;12:63.

S7. Khoury DS, Cromer D, Reynaldi A, et al. Neutralizing antibody levels are highly predictive of immune protection from symptomatic SARS-CoV-2 infection. Nat Med 2021;27:1205-1211.

S8. Perera RA, Mok CK, Tsang OT, et al. Serological assays for severe acute respiratory syndrome coronavirus 2 (SARS-CoV-2), March 2020. Euro Surveill 2020;25:2000421.
